# Clinical performance of metagenomic next-generation sequencing for diagnosis of pulmonary *Aspergillus* infection and colonization

**DOI:** 10.3389/fcimb.2024.1345706

**Published:** 2024-03-28

**Authors:** Ziwei Jiang, Wei Gai, Xiaojing Zhang, Yafeng Zheng, Xuru Jin, Zhiqiang Han, Geriletu Ao, Jiahuan He, Danni Shu, Xianbing Liu, Yingying Zhou, Zhidan Hua

**Affiliations:** ^1^ Department of Pulmonary and Critical Care Medicine, The Quzhou Affiliated Hospital of Wenzhou Medical University, Quzhou People’s Hospital, Quzhou, China; ^2^ WillingMed Technology (Beijing) Co., Ltd, Beijing, China

**Keywords:** pulmonary aspergillosis, *Aspergillus*, colonization, metagenomic next-generation sequencing, optimal threshold value

## Abstract

**Background:**

Investigations assessing the value of metagenomic next-generation sequencing (mNGS) for distinguish *Aspergillus* infection from colonization are currently insufficient.

**Methods:**

The performance of mNGS in distinguishing *Aspergillus* infection from colonization, along with the differences in patients’ characteristics, antibiotic adjustment, and lung microbiota, were analyzed.

**Results:**

The abundance of *Aspergillus* significantly differed between patients with *Aspergillus* infection (n=36) and colonization (n=32) (*P* < 0.0001). Receiver operating characteristic (ROC) curve result for bronchoalveolar lavage fluid (BALF) mNGS indicated an area under the curve of 0.894 (95%CI: 0.811-0.976), with an optimal threshold value of 23 for discriminating between *Aspergillus* infection and colonization. The infection group exhibited a higher proportion of antibiotic adjustments in comparison to the colonization group (50% vs. 12.5%, *P* = 0.001), with antibiotic escalation being more dominant. Age, length of hospital stay, hemoglobin, cough and chest distress were significantly positively correlated with *Aspergillus* infection. The abundance of *A. fumigatus* and Epstein-Barr virus (EBV) significantly increased in the infection group, whereas the colonization group exhibited higher abundance of *A. niger*.

**Conclusion:**

BALF mNGS is a valuable tool for differentiating between colonization and infection of *Aspergillus*. Variations in patients’ age, length of hospital stay, hemoglobin, cough and chest distress are observable between patients with *Aspergillus* infection and colonization.

## 1 Introduction

Pulmonary aspergillosis is the leading pulmonary fungal infection worldwide and a significant cause of morbidity and mortality (Bongomin et al., 2017). *Aspergillus* is present ubiquitously in the environment and has a high propensity for colonizing the human respiratory tract (Denning and Chakrabarti, 2017; El-Baba et al., 2020; Ledoux et al., 2020). The occurrence of fungal colonization does not result in disease immediately. However, it is a prerequisite for chronic and allergic mycoses, as well as for localized airway infections in invasive mycoses (Denning et al., 2014; Kosmidis and Denning, 2015). Various methods have been employed to detect fungi, including fungal culture, galactomannan (GM) antigen testing, 1, 3-β-D-glucan (G) testing, and specific polymerase chain reaction (PCR), etc (Mengoli et al., 2009; Shin et al., 2014; Boch et al., 2016; Lamoth et al., 2021; Lamoth and Calandra, 2022). However, identifying fungal infections is a considerable challenge, particularly distinguishing between fungal colonization and infection.

Currently, the use of metagenomic next-generation sequence (mNGS) technology is increasingly prevalent in the clinical diagnosis of infectious diseases, particularly when the etiology is not confirmed by conventional microbiological testing (CMT) or when anti-infective treatment proves ineffective. Several studies have demonstrated that mNGS is highly effective in diagnosing mixed infections, and viruses’ infections (Carpenter et al., 2019; Reyes et al., 2021; Mei et al., 2023; Ogunbayo et al., 2023; Xie et al., 2023). Compared to culture and other CMT methods, mNGS has a higher positive rate, sensitivity, and specificity in diagnosing invasive fungal infections (IFA) (Song et al., 2021; Li, 2022; Wang et al., 2022). In some cases, it can even detect fungal pathogens that are difficult to diagnose using conventional methods (Liu et al., 2022; Chen et al., 2023a). Furthermore, research has investigated the diagnostic utility of mNGS in non-neutropenic patients, those with COVID-19, and immunocompromised patients with pulmonary *Aspergillus* infection (Bao et al., 2022; Hoenigl et al., 2023; Zhan et al., 2023). However, there is still a dearth of studies on differentiating between *Aspergillus* infection and colonization.

Nevertheless, recent studies using mNGS to differentiate between fungal colonization and infection have emerged. Moreover, these studies have focused on exploring the threshold of mNGS read numbers for distinguishing between infection and colonization/not infection. The study of Liu et al. revealed that bronchoalveolar lavage fluid (BALF) mNGS can distinguish between *Pneumocystis jirovecii* colonization and infection with an area under the curve of 0.973, and an optimal threshold value of 14 reads (Liu et al., 2021). Jia et al. discovered that the optimal cut-off value for BALF mNGS in diagnosing invasive pulmonary aspergillosis (IPA) and no-IPA was species-specific read number (SSRN) is 2.5. Additionally, they found that thresholds of 1 and 4.5 was appropriate for immunocompromised and diabetic patients with IPA, respectively (Jia et al., 2023). Studies on the clinical characteristics of patients, as well as the differences in the microbial composition of the lungs of patients with *Aspergillus* colonization and infection, remains inadequate.

In this study, we evaluated the effectiveness of mNGS and conventional microbiological testing (CMT) for distinguished patients with *Aspergillus* infection from those with colonization. We outlined the antibiotic guidance between the two groups, the variations in clinical indicators, as well as changes in the microbial composition of the lungs.

## 2 Methods

### 2.1 Patients and study design

We conducted a retrospective study at the Quzhou People’s Hospital, including patients who were admitted between August 2021 and June 2023 with suspected fungal pulmonary infections and underwent BALF mNGS detection. The study was approved by the Ethics Committee of Quzhou People’s Hospital (2023-017), and the patients were anonymized for analysis. The corresponding medical records were reviewed, and the clinical data analyzed including demographic characteristics, type of underlying disease, diagnosis, clinical course, treatment, and outcome.

BALF, blood, sputum and some other types of respiratory tract samples were used for pathogen identification through CMT methods, including culture for bacteria (blood agar plates, Chocolate, and MacConkey) and fungi (Sabouraud agar plates), 1, 3-β-D-glucan (G) test (Fungi (1,3)-β-D-glucan assay kit, Ref#KTG20, BIOENDO) and galactomannan (GM) test (Platelia *Aspergillus* Ag Assay, Ref#62794, Bio-Rad) for fungi, serum antigen detection for *Cryptococcus* (Ref#CR2003, IMMY), T-spot (Ref#30230808, WANTAI Biopharm) and Gene X-pert (Ref#20173406215, Cepheid AB) for *Mycobacterium*, and smear microscopy for fungi (fluorescent and KOH stain) or tuberculosis (acid-fast stain). All BALF samples were cultured for bacteria and fungi identification.

### 2.2 mNGS detection

BALF specimens were collected by experienced bronchoscopist under general anesthesia following standard procedures. Lesions were selected based on computed tomography images of the chest. Following general anesthesia with sufentanil and propofol (to ensure a smooth bronchoscopic procedure and prevent potential patient discomfort-related injuries), the patient was guided to the target bronchial location through the contralateral nasal cavity. Nasopharyngeal ventilation and electrocardiographic monitoring were used during the procedure. No suction was performed before entering the target location. The fiberoptic bronchoscope’s tip was tightly wedged into the opening of the target bronchial segment or subsegment. Then, 100-250 mL of 37°C sterilized saline was rapidly injected through the silicone tube via the biopsy hole. The saline was divided into 3-5 equal parts, with each part being 25-60 mL. After each perfusion, 50-100 mmHg (1mmHg=0.133kPa) negative pressure suction was used to recover the irrigation fluid. The BALF (3-5 mL) obtained from the second perfusion from each patient was collected into sterile tubes according to standard procedures and transported at 4-8 °C within a short time after collection. DNA was extracted using PathoXtract^®^ Basic Pathogen Nucleic Acid Kit (WYXM03211S, WillingMed Corp, Beijing, China) according to the manufacturer’s protocol. DNA libraries was constructed using the Illumina^®^ DNA Prep, (M) Tagmentation kit (20018705, Illumina). Quality control of the DNA libraries was conducted using the Agilent 2100 Bioanalyzer (Agilent Technologies) to ensure library concentration exceeded 1 ng/μL. Then, libraries with confirmed quality were sequenced by the NextSeq™ 550Dx platform with a 75 bp, single-end sequencing kit (Illumina). To exclude environmental and laboratory contaminants, an external negative control (nuclease-free water) was included in each mNGS assay run (Blauwkamp et al., 2019).

High-quality sequencing data were generated by filtered out low-quality or undetected sequences, sequences contaminated by splices, high-coverage repeats, and short-read-length sequences using Trimmomatic v0.40. The remaining sequences were aligned to four databases downloaded from NCBI, including bacteria, fungi, viruses, and parasites, for classification after removal of human host sequences with Bowtie2 v2.4.3. For identification of the pathogens, a RPTM (reads per ten million) value, which defined as detected number of pathogen specific reads per ten million, was used. The RPTM ratio metric (RPTM-r), defined as RPTM_Sample_/RPTM_NTC_, set to 1 in NTC samples when the microbial taxonomic value of a given species or genus is less than 1 (Miller et al., 2019). RPTM ≥ 3 was used as an empirical threshold for virus detection. For bacteria and fungi, positive pathogens were required to meet a RPTM threshold ≥ 20 (Chen et al., 2023b). Special pathogens (including *Cryptococcus* and *Mycobacterium*) with RPTM ≥ 1 was identified as positive (Langelier et al., 2018; Chen et al., 2021). Common colonized bacteria and contaminating microorganisms, including *Rothia*, *Corynebacterium*, coagulase-negative *Staphylococci*, *Neisseria*, and *Streptococcus* etc., were considered as microecological bacteria in the study (Chen et al., 2020; Xie et al., 2021; Shi et al., 2022; Tan et al., 2023).

### 2.3 Criteria for *Aspergillus* infection diagnosis

We applied the criteria of the European Organization for Research and Treatment of Cancer and Mycoses Study Group (EORTC/MSG) for diagnose of invasive pulmonary aspergillosis (IPA) and Chronic necrotizing pulmonary aspergillosis (CNPA) (Donnelly et al., 2020). Proven IPA diagnosis criteria: histopathology, cytopathology, or direct microscopy of specimens obtained by needle aspiration or biopsy revealed hyphae, and accompanied by evidence of associated tissue damage. Alternatively, molds are cultured by aseptic procedures from specimens obtained from normally sterile, clinically, or radiologically abnormal sites consistent with an infectious disease process. Probable *Aspergillus* infection is definite as the presence of at least one host factor, a clinical feature and mycologic evidence. Possible cases meet the criteria of with a host factor and a clinical feature, but for which mycological evidence has not been found. The diagnostic criteria for chronic pulmonary aspergillosis (CPA) are based on the 2016 clinical guidelines from the European Society for Clinical Microbiology and Infectious Diseases (ESCMID) and the European Respiratory Society (ERS) (Denning et al., 2016).

Regarding the criteria for *Aspergillus* positivity in the CMT methods, positive culture results represent *Aspergillus* recovered by culture from a sputum, BALF or bronchial brush sample. A positive GM result is definite as a value ≥ 1.0 (Donnelly et al., 2020). Proven and probable cases were classified into *Aspergillus* infection group (Feys et al., 2022). Patients with positive mycological cultures or amplification of fungal DNA without signs of infection were classified as *Aspergillus* colonization (Donnelly et al., 2020). Therefore, cases were considered as colonization when *Aspergillus* was identified but without a final diagnosis of *Aspergillus* infection. Two experienced physicians made clinical diagnoses; when they gave different results, another senior physician made a judgement.

### 2.4 Statistical analysis

The chi-square test was applied to the categorical variables. A student *t*-test was used for continuous variables. *P*-value less than 0.05 was considered statistically significant. All statistical analyses were performed using GraphPad Prism (Version 9.4.1, GraphPad Software Inc) and SPSS (version 26, IBM Corp). The diagnostic performance of mNGS was evaluated using the area under the curve (AUC) of Receiver operating characteristic (ROC), where the best cut-off values were obtained. The sensitivity and specificity of the detection method were analyzed as reference (Blauwkamp et al., 2019). The alpha diversity index was calculated based on Shannon and Simpson indexes. Beta-diversity was visualized using principal coordinate analysis (PCoA), and an ANOSIM test was performed in R with the Vegan package. The stacked bar plot of the community composition was visualized in R using the ggplot2 package. Linear discriminant analysis (LDA) effect size (LEfSe) was utilized to identify significantly different species among the groups, with thresholds of log10 LDA Score ≥ 2 and *P* value ≤ 0.05.

## 3 Results

### 3.1 Baseline characteristics and sample classification

**Figure 1:**
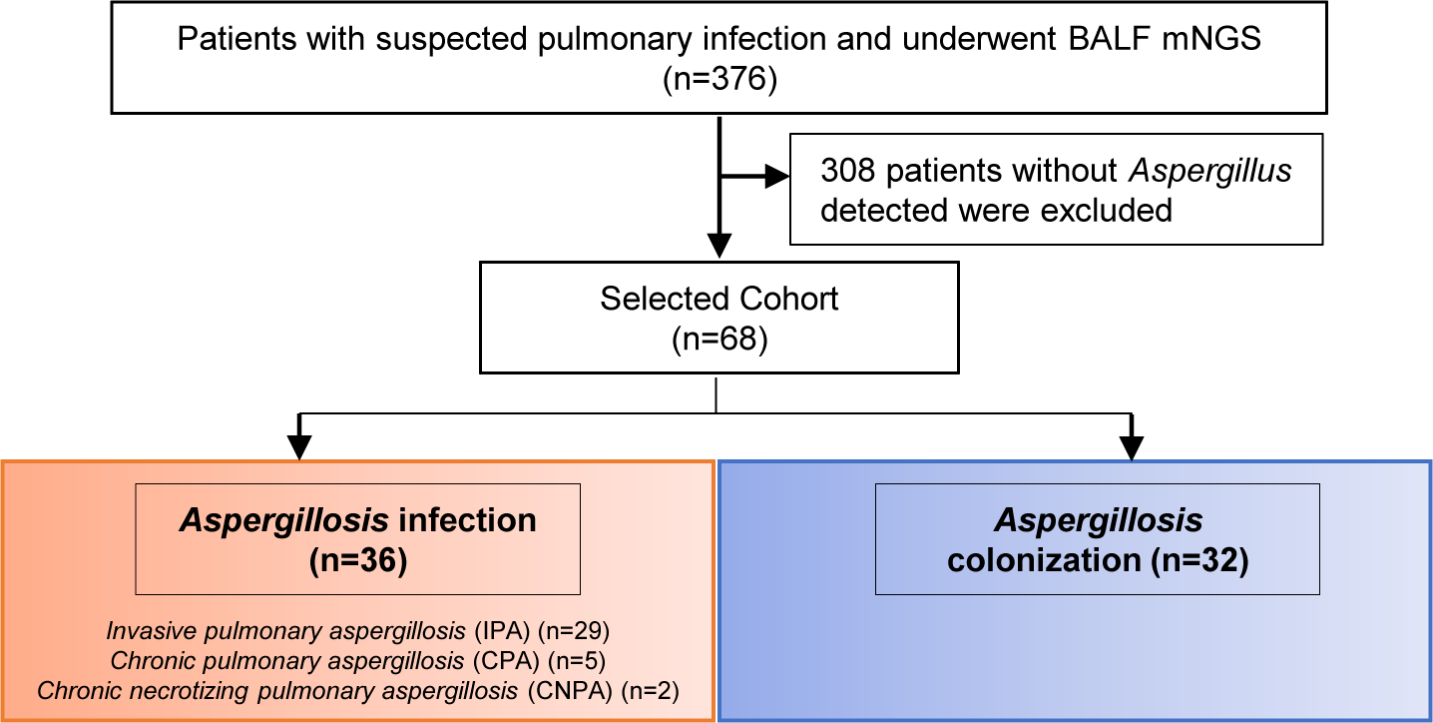
Flow diagram of the patients included in the study.

A total of 68 patients were screened from a cohort of 376 individuals with suspected pulmonary infection who underwent BALF mNGS. Among them, 36 were diagnosed with *Aspergillus* infection and 32 were identified as cases of *Aspergillus* colonization. Within the group of patients with *Aspergillus* infection, 29 were diagnosed with invasive pulmonary aspergillosis (IPA, all proven cases), 5 with chronic pulmonary aspergillosis (CPA), and 2 with chronic necrotizing pulmonary aspergillosis (CNPA, 1 proven and 1 probable) (**Figure 1**).

Baseline data was compared between patients with *Aspergillus* infection and colonization. The patients’ ages ranged from 21 to 89 years, with a median age of 65. Of the 68 patients, 60.29% (41/68) were male. Those with *Aspergillus* infection were found to be significantly older than those with *Aspergillus* colonization (68.44 ± 11.66 vs. 61.53 ± 14.35, *P* = 0.032). Cough and chest distress symptoms were more frequently observed in patients with *Aspergillus* infection (*P* = 0.021, and *P* = 0.020). Additionally, patients with *Aspergillus* infection had a longer length of hospital stays (LOHS) compared with those with *Aspergillus* colonization (10.76 vs. 7.22 days, *P* = 0.011). There was no significant difference in terms of the proportion of patients who were immunosuppressed or had malignant tumors between the two groups. Pulmonary infection, pneumonia, and tuberculosis were the prevalent diseases observed in patients (**Table 1**). Patients with *Aspergillus* infection had a lower hemoglobin (Hb) level than patients with *Aspergillus* colonization (*P* = 0.027). Patchy shadow, pulmonary emphysema, pleural effusion, and nodules were common lung imaging findings that did not differ significantly between groups (**Table 2**).

**Table 1:** General characteristics of the patients with *Aspergillus* infection and colonization.

Characteristic ^a^	All patients (n=68)	*Aspergillus* infection (n=36)	*Aspergillus* colonization (n=32)	*P*-value ^b^
Age, mean ± SD (Year)	65.19 ± 13.36	68.44 ± 11.66	61.53 ± 14.35	0.032*
Gender (Male)	41 (60.29%)	21 (58.33%)	20 (62.5%)	0.726
Underlying condition				
Hypertension	11	4	7	0.229
Diabetes mellitus	2	0	2	0.128
Malignant tumor	11	7	4	0.438
Liver disease	9	6	3	0.376
Renal disease	5	4	1	0.208
Cardiovascular disease	15	10	5	0.228
COPD	4	2	2	0.903
Bronchiectasis	11	5	6	0.587
Symptoms				
Fever	16	7	9	0.400
Cough	53	32	21	0.021*
Expectoration	50	28	22	0.400
Chest distress	27	19	8	0.020*
Hemoptysis	7	3	4	0.573
Immunocompromised	9	7	2	0.109
LOHS (day)	9.03 ± 5.62	10.64 ± 6.82	7.22 ± 3.08	0.011*
Final diagnoses				
Severe pneumonia	5	4	1	0.208
Pneumonia	8	4	4	0.859
Pulmonary infection	53	27	26	0.535
AECOPD	3	2	1	0.626
Pulmonary abscess	2	2	0	0.176
Pulmonary tuberculosis	6	3	3	0.880

a COPD, chronic obstructive pulmonary disease; LOHS, length of hospital stays; AECOPD, acute exacerbation of chronic obstructive pulmonary disease.

b Analysis of significant differences between baseline data of *Aspergillus* infection and colonization patients. * Indicated that the *P*-value < 0.05.

**Table 2:** Comparison of clinical test and chest computed tomography results of the patients with *Aspergillus* infection and colonization.

Characteristic ^a^	*Aspergillus* infection (n=36)	*Aspergillus* colonization (n=32)	*P* – value
Clinical test			
CRP (µg/mL)	58.20 ± 75.80	32.86 ± 47.96	0.109
WBC (× 10 ^9/^L)	9.81 ± 18.33	6.57 ± 2.62	0.325
RBC (× 10 ^9/^L)	3.81 ± 0.88	3.93 ± 0.62	0.518
NEUT%	68.44 ± 20.77	67.93 ± 13.16	0.906
LY%	19.14 ± 13.35	21.49 ± 10.62	0.428
Hb (g/L)	107.53 ± 18.57	118.13 ± 19.97	0.027*
PLT (× 10 ^9/^L)	187.94 ± 92.95	182.69 ± 65.18	0.790
Cr (µmol/L)	73.17 ± 43.06	68.16 ± 21.01	0.552
BUN (mmol/L)	7.68 ± 12.72	5.00 ± 2.48	0.245
TBIL (µmol/L)	10.46 ± 6.91	13.77 ± 8.95	0.091
ALT (U/L)	37.08 ± 45.27	23.45 ± 18.53	0.122
LDH (U/L)	269.55 ± 150.40	232.33 ± 102.65	0.243
CT findings			
Nodules	9	7	0.762
Consolidation	0	2	0.128
Atelectasis	2	1	0.626
Ground glass shadow	1	2	0.487
Tree in bud	0	2	0.128
Cavities	1	3	0.249
Patchy shadow	14	9	0.349
Pulmonary emphysema	12	9	0.643
Pleural effusion	9	8	>0.999

a CRP, C-reactive protein; WBC, white blood cell; RBC, red blood cell; NEUT, neutrophil; LY, lymphocyte; Hb, hemoglobin; PLT, platelet; Cr, creatinine; BUN, blood urea nitrogen; TBIL, total bilirubin; ALT, alanine aminotransferase; LDH, lactate dehydrogenase.

*Indicated that the *P*-value < 0.05.

**Figure 2:**
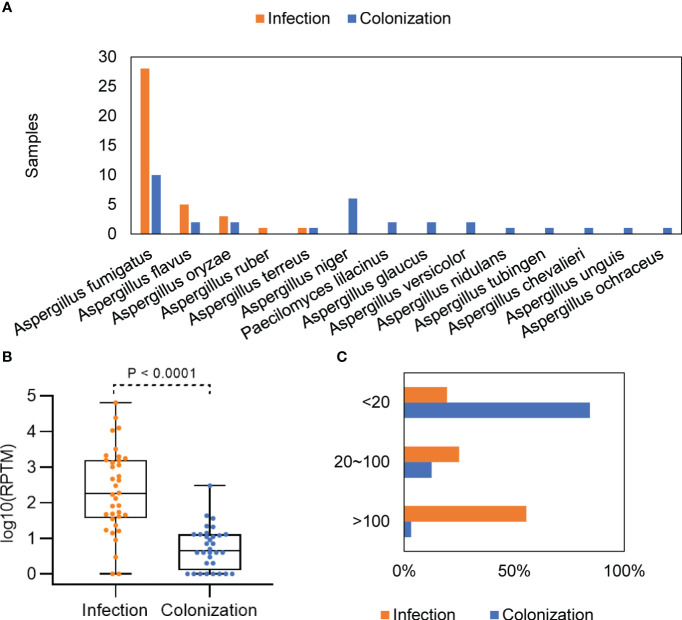
Distribution and abundance of *Aspergillus* species in patients with *Aspergillus* infection and colonization. **(A)** Comparison of *Aspergillus* in patients with *Aspergillus* infection and colonization. **(B)** Differences in mNGS RPTM for *Aspergillus* in patients with *Aspergillus* infection and colonization. **(C)** Proportion of patients with different mNGS *Aspergillus* reads in the infection and colonization groups.

In the group of *Aspergillus* infection and colonization, mNGS identified of 5 and 13 *Aspergillus* species, respectively (**Supplementary Table 1**). Among patients with *Aspergillus* infection, *A. fumigatus* (77.78%, 28/36) was the most common species, followed by *A. flavus* (13.89%, 5/36) and *A. oryaze* (5.56%, 2/36). One patient was found to be infected with both *A. flavus* and *A. oryzae*. In patients with *Aspergillus* colonization, *A. fumigatus* (31.25%, 10/32) and *A. niger* (18.75%, 6/32) were the most detected species (**Figure 2A**).

The *Aspergillus* load was significantly higher in the group with *Aspergillus* infection compared with the group with colonization, with a median mNGS read number of 3609 ± 11358 vs. 17.88 ± 52.44 (*P* < 0.0001) (**Figure 2B**). When patients were divided into low (≤ 20), media (20 ~ 100), and high (≥ 100) groups based on the mNGS RPTM value for *Aspergillus*, the results showed that over 80% of patients in the infection group had an RPTM value larger than 20, while over 80% of patients in the colonization group had an RPTM value less than 20 (**Figure 2C**).

### 3.2 Diagnostic efficacy of BALF mNGS for *Aspergillus* infection and colonization

**Figure 3:**
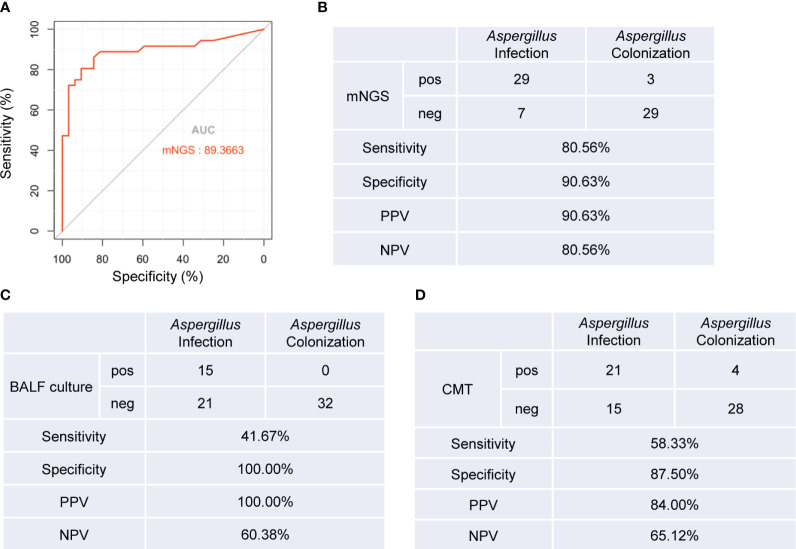
Diagnostic performance of mNGS, BALF culture and CMT tests for distinguishing *Aspergillus* infection from colonization. **(A)** ROC curve of mNGS for discrimination between *Aspergillus* infection and colonization. **(B-D)** Diagnostic performance of mNGS **(B)**, BLAF culture **(C)**, and CMT **(D)** methods for differentiating between *Aspergillus* infection and colonization. AUC, area under curve. PPV, positive predictive value; NPV, negative predictive value. pos, positive; neg, negative.

We generated an ROC curve using the *Aspergillus* RPTM of mNGS in BALF from the patients. The calculated area under curve (AUC) was 0.894 (95% CI: 0.811-0.976), with the optimal cut-off value for distinguishing *Aspergillus* infection from colonization was determined to be 23 (**Figure 3A**).

Next, we evaluated the diagnostic accuracy of BALF mNGS, BALF culture, and conventional microbiological testing (CMT) (**Supplementary Table 1**) in distinguishing *Aspergillus* infection from colonization. Using RPTM ≥ 23 as the threshold criterion for *Aspergillus* infection, mNGS demonstrated a sensitivity of 80.56%, which was significantly higher than BALF culture (41.67%, *P* = 0.001), and CMT methods (58.33%, *P* = 0.041). There was no significant difference in specificity between mNGS and BALF culture (90.63% vs. 100%, *P* = 0.076), nor between mNGS and CMT methods (90.63% vs. 87.5%, *P* = 0.689). However, the specificity of BALF culture was significantly higher than that of CMT methods (*P* = 0.039) (**Figures 3B-D**).

### 3.3 Impacts of mNGS on antibiotic usage of *Aspergillus* infection patients

**Figure 4:**
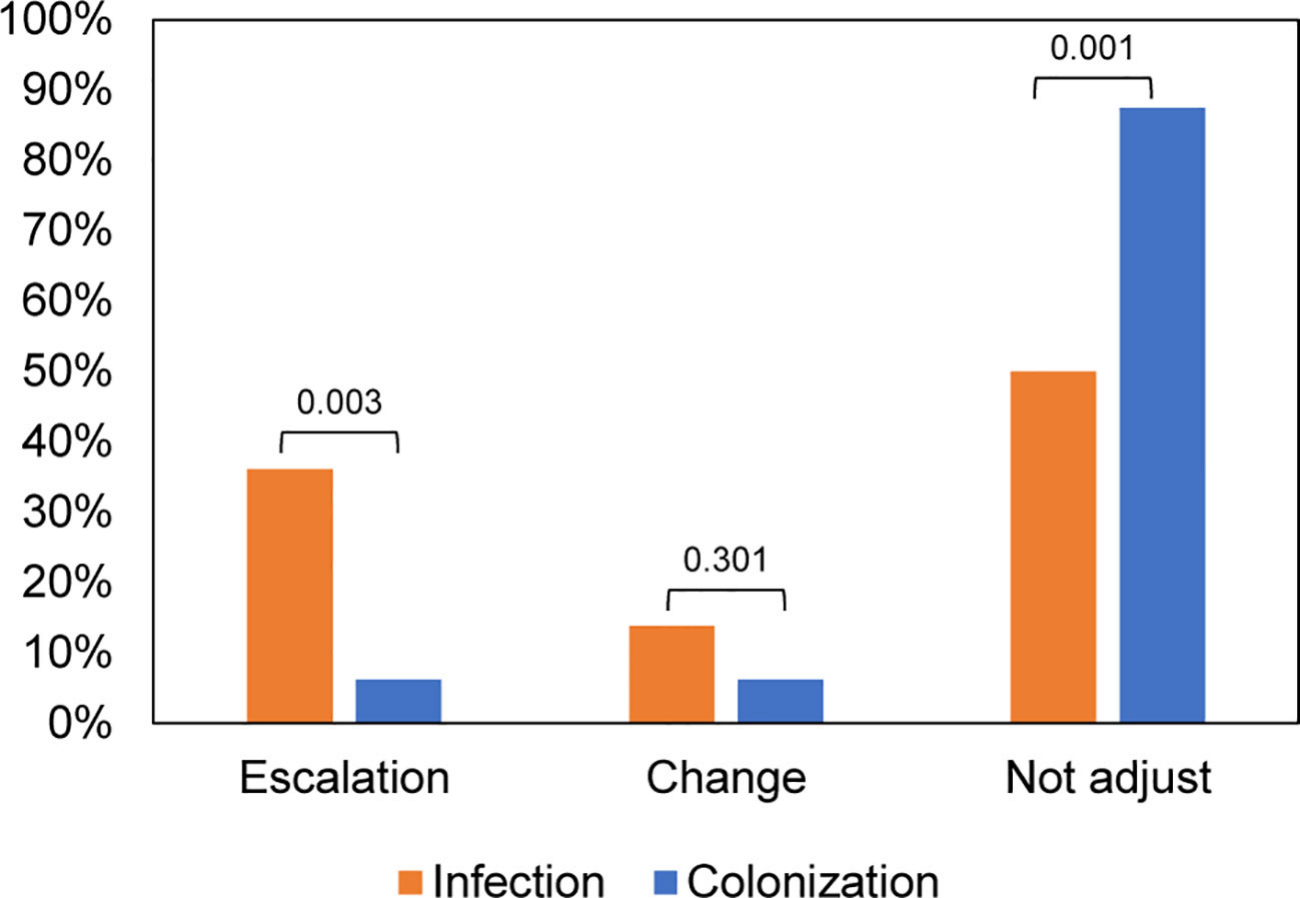
Impacts of mNGS on antibiotic adjustment in patients with *Aspergillus* infection and colonization.

To explore the influence of mNGS results on antimicrobial usage, we analyzed variations in antimicrobial regimens before and after mNGS detection. The patients in the *Aspergillus* infection group had a higher incidence of combined bacteria and fungi infection (69%, 25/36), whereas the predominant infection in the *Aspergillus* colonization group was bacterial (78%, 25/32) (**Supplementary Figure 1**). We found that antimicrobial drug regimens were adjusted in 18 out of 36 (50%) samples from patients with *Aspergillus* infection, which was significantly higher than the proportion of patients with *Aspergillus* colonization (12.5%, *P* = 0.001). Among the 18 samples, 5 cases had their antibiotics changed, while 13 samples had their antibiotics escalated. The percentage of patients requiring antibiotic escalation was significantly higher in the *Aspergillus* infection group compared to the *Aspergillus* colonization group (*P* = 0.003) (**Figure 4**).

### 3.4 Correlations between the characteristics and *Aspergillus* infection

**Figure 5:**
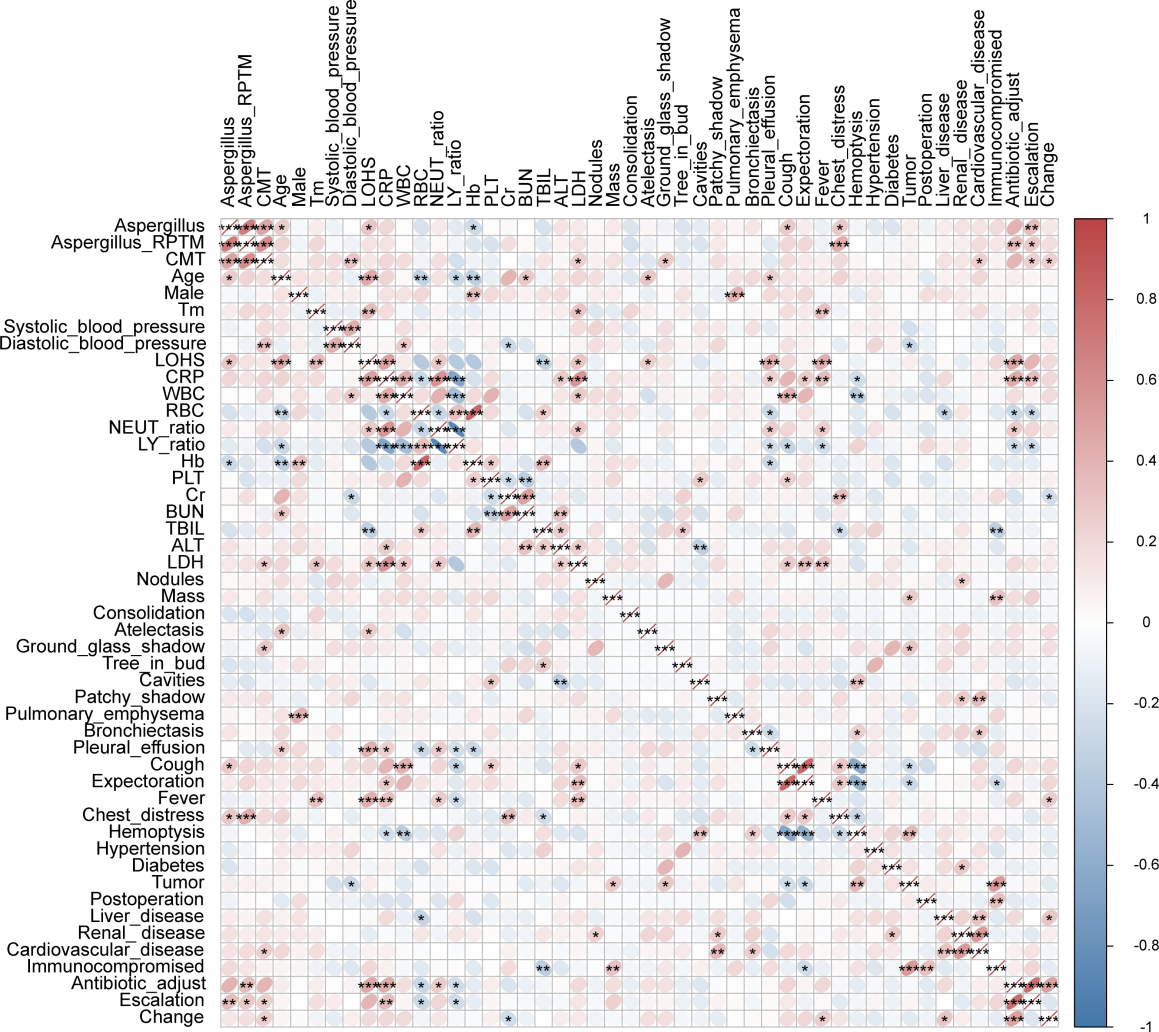
Spearman correlations between *Aspergillus* infection and characteristics of patients. Tm, temperature. “Change” indicates the adjustment of antibiotic. * *P* < 0.05, ** *P* < 0.01, *** *P* < 0.001.

We conducted Spearman correlation analyses to examine the relationship between various characteristics and *Aspergillus* infection. The results showed significant positive correlations between *Aspergillus* infection and the following variables: *Aspergillus* RPTM, CMT *Aspergillus* test results, age, LOHS, presence of cough and chest distress symptoms, and antibiotic escalation. And significant negative correlations between *Aspergillus* infection and Hb level were observed. Additionally, significant positive correlations were observed between *Aspergillus* RPTM values and the following variables: CMT *Aspergillus* test results, chest distress symptoms, and antibiotic adjustments and escalation. Notable, CMT *Aspergillus* positivity was positively correlated with diastolic blood pressure, lactate dehydrogenase (LDH), ground-glass shadow, presence of cardiovascular disease in the patient, and antibiotic escalation and change. Additionally, positive correlations were found between antibiotic adjustments and LOHS, CRP levels, and NEUT%. However, antibiotic adjustments were significantly negatively correlated with RBC and LY% (**Figure 5**).

### 3.5 Differences in the microbial community structure

**Figure 6:**
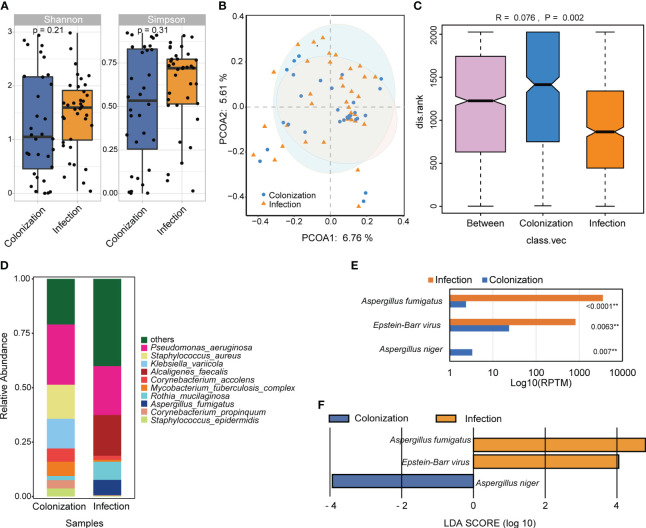
The difference of microbial composition for patients with *Aspergillus* infection and colonization. **(A)** Alpha diversity was showed by Shannon and Simpson index. **(B)** PCoA analysis of the microbial composition. **(C)** ANOSIM for the analysis of microbial community structure. **(D)** Barplot showed the top 10 species with the highest abundance between two groups. **(E)** Significant different analysis of the species between two groups with Kruskal-Wallis test. **(F)** Lefse analysis for enriched species for the two groups. ** *P* < 0.01.

The study compared the overall composition and diversity of the lung microbial signature in patients with *Aspergillus* infection and colonization. Although no significant difference was observed, patients with *Aspergillus* infection showed a higher α diversity according to both the Shannon and Simpson indices, indicating a trend towards increased richness and evenness of microbial composition (**Figure 6A**). Principal co-ordinates analysis (PCoA) results indicated that the samples from both groups were intermixed. However, the colonization group displayed a wider spread of data compared to the infection group (**Figure 6B**). Moreover, a significant difference in the microbial community structure between the two groups was observed (**Figure 6C**).

The relative abundance of the top 10 species were examined, which included *Pseudomonas aeruginosa*, *Staphylococcus aureus*, *Klebsiella variicola*, *Alcaligenes faecalis*, *Corynebacterium accolens*, *Mycobacterium tuberculosis* complex, *Rothia mucilaginosa*, *Aspergillus fumigatus*, *Corynebacterium propinquum*, and *Staphylococcus epidermidis*. Among these species, only *A. fumigatus* showed significant differences between the two groups (**Figures 6D, E**). Additionally, three species with LDA scores ≥ 2 and a significance level of *P* < 0.05 were identified. *A. fumigatus* and Epstein-Barr virus (EBV) were significantly more abundant in patients with *Aspergillus* infection, whereas *A. niger* was more enriched in cases with *Aspergillus* colonization (**Figure 6F**).

## 4 Discussion

The prevalence of pulmonary aspergillosis has significantly increased in recent years and is associated with the high mortality rates (Bongomin et al., 2017). *Aspergillus* colonization may indicate a transient presence within the airway, as long-term non-threatening carriage is often identified in patients with restrictive structural or functional lung defects. However, it can serve as an early warning sign before the development of apparent *Aspergillus* infection (Soubani et al., 2004; Khasawneh et al., 2006). Timely and precise diagnosis of *Aspergillus* infection and colonization is crucial for effective treatment, but poses significant clinical challenges (Thompson and Young, 2021). Although *Aspergillus* infection and colonization have clear definitions (Donnelly et al., 2020), different diagnostic methods vary in their performance for identifying *Aspergillus* (Mengoli et al., 2009; Shin et al., 2014; Boch et al., 2016; Lamoth et al., 2021; Lamoth and Calandra, 2022). However, studies aimed at distinguishing between *Aspergillus* infection and colonization are still inadequate. This study evaluated the efficacy of mNGS in differentiating *Aspergillus* infection from colonization. Furthermore, it outlined the distributional characteristics of *Aspergillus*, the distinctions in clinical characteristics and antibiotic adjustment among patients with *Aspergillus* infection and colonization, as well as the variations in lung microbiota.

*Aspergillus* is a ubiquitous fungus found worldwide. Among the over 200 species in the genus *Aspergillus*, only a small percentage of them are known to infect humans (Nabili et al., 2016; Khojasteh et al., 2023). Notably, *A. fumigatus*, *A. flavus*, *A. niger*, and *A. terreus* are the most common species causing disease in humans (Sugui et al., 2014; Lamoth and Calandra, 2022). Consistent with previous studies, our study identified 14 *Aspergillus* species among the patients, with only 5 species leading to *Aspergillus* infection. Among these, *A. fumigatus*, *A. flavus*, and *A. oryaze* were the most prevalent in patients with *Aspergillus* infection. *A. niger*, except for *A. fumigatus*, was the most common species in patients with *Aspergillus* colonization (**Figure 2A**). Although *A. niger* only played a role only in colonization in our study, it has the potential to cause pneumonia. In a study of Japan, *A. niger* was also identified as the dominant species in patients with *Aspergillus* colonization, and it was most commonly isolated in patients with allergic bronchopulmonary aspergillosis (Tashiro et al., 2011). A case report described a chronically steroid-treated COPD patient presenting with hemoptysis and pleuritic chest pain, where the presence of calcium oxalate crystals in histopathological specimens was a typical sign of *A. niger* infection (Person et al., 2010). *A. flavus* was found to be more prevalent in certain regions of Africa or Asia (Lamoth and Calandra, 2022). Several cases of pulmonary infection caused by a combination of *A. flavus* and other pathogens have been identified. In one case, a 60-year-old man exhibited patchy high-density shadows and a few pleural effusions in the transplanted lung, indicating a mixed infection of *Klebsiella pneumoniae*, *Ureaplasma urealyticum*, and *A. flavus* (Liu et al., 2023). Another case involved a 62-year-old male with co-infection of *A. flavus* and *Klebsiella pneumoniae*. This patient exhibited leukopenia, thrombocytopenia, acute kidney failure, and severe myelosuppression (Xu et al., 2022).

In recent years, the benefits of using mNGS for fungal detection have become increasingly apparent. A meta-analysis has shown that mNGS has a combined sensitivity and specificity of 78% (95% confidence interval [CI]: 67-87%; I^2^ = 92%) and 77% (95% CI: 64-94%; I^2^ = 74%), respectively, for fungal diagnosis. Subgroup analysis revealed that the sensitivity of mNGS was significantly affected by severe or immunocompromised patients with pulmonary infection (*P <* 0.001) (Chen et al., 2022). This suggests that mNGS testing, even before CMT results are available, is a highly effective option. Early detection of pathogenic microorganisms in patients with severe infections is crucial for providing appropriate clinical intervention and targeted antibiotics as soon as possible. There are many reasons for this heterogeneity of mNGS for fungi identification, one of which may be that the thick cell wall of *Aspergillus* makes it difficult to break the wall and release nucleic acid, which may cause false negative mNGS results (Bittinger et al., 2014). However, the diagnostic performance of mNGS for *Aspergillus* has improved with the optimization of DNA extraction methods (Gu et al., 2021). Additionally, the positive threshold criteria for mNGS may also be an important influencing factor (Chen et al., 2022). Thus, it is crucial to determine the positive threshold of mNGS in clinical applications, but there is currently no unified international standard. Our study has laid the foundation for the establishment of this standard to some extent.

Our study found that BALF mNGS demonstrated superior accuracy in diagnosing *Aspergillus* infection and distinguishing it from colonization when compared to BALF culture and CMT methods (*P* < 0.05). The optimal cut-off value of RPTM for mNGS was 23. At this threshold, mNGS achieves a sensitivity of 80.56% and a specificity of 90.63% for the final diagnosis (**Figure 3**). While there have been numerous studies investigating the diagnostic ability of mNGS for *Aspergillus* infection, there remains a lack of research regarding the discrimination between *Aspergillus* colonization and infection (Jia et al., 2023). In addition, the value of Bio-Rad Platelia *Aspergillus* IgG (Platelia *Aspergillus* IgG) enzyme-linked immunosorbent assay (ELISA) method and the *Aspergillus* precipitin test for distinguish pulmonary aspergillosis from colonization were assessed. Platelia *Aspergillus* IgG had a higher positive rate than *Aspergillus* precipitin test for *Aspergillus* detection, and showed a sensitivity of 74.5% and a specificity of 94.8% for distinguishing *Aspergillus* infection from colonization (Shinfuku et al., 2023). Their diagnostic accuracy was slightly lower than that of mNGS. Nevertheless, the current research serves as a valuable reference for analyzing patients with *Aspergillus* colonization and infection. While mNGS serves as a precise pathogenetic test and holds potential in clinical diagnosis, the final diagnosis of the disease depends on clinical experts who integrate the patient’s symptoms, clinical test results, and pathogenetic findings to make a comprehensive decision.

We examined the clinical characteristics of patients with *Aspergillus* infection and colonization and found that patients with *Aspergillus* infection were older and had longer lengths of hospital stay (**Table 1**). This trend was also found in COVID-19 patients with or without fungal coinfection (Negm et al., 2023). Common non-specific symptoms observed in patients with *Aspergillus* infection included persistent fever, dry cough, hemoptysis, and chest pain (Guo et al., 2023). In this study, cough, expectoration, and chest distress were found to be more prevalent, and the proportion of patients experiencing cough and chest distress symptoms was higher in the *Aspergillus* infection group. Although immunosuppression is a crucial risk factor for *Aspergillus* infections (Patterson et al., 2016), there was no significant difference in immunosuppression status between the two groups (**Table 1**). Among routine blood indicators, only hemoglobin levels showed significant differences, with a marked increase in the *Aspergillus* colonization group compared to the infection group. CT findings indicated that *Aspergillus* infection and colonization were characterized by patchy shadows, pulmonary emphysema, pleural effusion, and nodules (**Table 2**). Correlation analysis confirmed the significance of these characteristics in relation to *Aspergillus* infection and colonization. In addition, a significant positive correlation was observed between *Aspergillus* infection and mNGS determined antibiotic adjustment (**Figure 5**). We observed no significant differences in species abundance and diversity between the groups with *Aspergillus* infection and colonization, although these indexes were higher in the infection group (**Figure 6**). *A. fumigatus* and EBV appeared more frequently in patients with *Aspergillus* infection. EBV is a member of the gamma herpesviruses and is one of the most common human viruses. During childhood, all people are infected with multiple herpesviruses, and after clearing an acute infection, herpesviruses enter a latent state (Barton et al., 2007). EBV is commonly associated with infectious mononucleosis, lymphoma, and cancer in adolescents and adults (Epstein et al., 1964; Ok et al., 2015). However, cases of pneumonitis due to EBV infection in adults are rare (McManus et al., 2009; Niazi et al., 2020; Liao et al., 2023). In our study, only two immunocompetent patients were diagnosed with EBV infection, one with *A. fumigatus* and one with *A. oryzae*. In addition, EBV was also detected in another 12 patients with *Aspergillus* infection and 4 patients with *Aspergillus* colonization in this study. A previous study observed a significantly different microbial structure between IPA and non-IPA patients, with cytomegalovirus and *A. fumigatus* appeared more frequently in patients with IPA (Ao et al., 2023). Further exploration is necessary to understand the relationship and potential interaction mechanism between the two pathogens. Several factors may account for this disparity of the different results of microbiome analysis, including the advanced age of our patients, their relatively low mortality rate, and the absence of any restrictions on the type of diseases they presented.

In this investigation, we conducted a comprehensive study to analyze the clinical characteristics and lung microbiota changes in individuals with *Aspergillus* infection and colonization. Moreover, we evaluated the efficacy of BALF mNGS in distinguishing between *Aspergillus* infection and colonization. While our study was meticulously designed and analyzed, we acknowledge its limitations. Bacterial and fungal cultures of BALF were performed in all patients, but not all patients underwent G/GM tests, resulting in a lack of corresponding comparative diagnostic performance results. Furthermore, this study was conducted in a single-center and the sample collection was limited to a two years period, which may introduce bias in the outcomes.

## 5 Conclusions

In this study, the performance of mNGS in distinguishing *Aspergillus* infection from colonization, along with the differences in patients’ characteristics, antibiotic adjustment, and lung microbiota, were analyzed. We found that BALF mNGS has a high diagnostic efficacy for distinguishing *Aspergillus* infection and colonization, which was superior to BLAF culture and the CMT methods used in this study. Furthermore, mNGS plays a more important role in the guidance of medication in patients with *Aspergillus* infection. Age, LOHS, cough, chest distress, and Hb were significant different indicators between patients with *Aspergillus* infection from colonization.

## Data availability statement

The original contributions presented in the study are included in the article/**Supplementary Materials**, further inquiries can be directed to the corresponding author/s.

## Ethics statement

The studies involving humans were approved by the Ethics Committee of Quzhou People’s Hospital. The studies were conducted in accordance with the local legislation and institutional requirements. The ethics committee/institutional review board waived the requirement of written informed consent for participation from the participants or the participants’ legal guardians/next of kin because Written informed consent was waived by the ethics committee due to the retrospective nature of study design. We analyzed the data anonymously.

## Author contributions

ZJ: Conceptualization, Data curation, Formal analysis, Visualization, Writing – original draft, Writing – review & editing. WG: Conceptualization, Formal analysis, Supervision, Validation, Visualization, Writing – original draft, Writing – review & editing. XZ: Conceptualization, Data curation, Formal analysis, Visualization, Writing – original draft, Writing – review & editing. YaZ: Conceptualization, Formal analysis, Supervision, Visualization, Writing – original draft, Writing – review & editing. XJ: Data curation, Resources, Supervision, Writing – review & editing. ZH: Data curation, Resources, Supervision, Writing – review & editing. GA: Data curation, Resources, Supervision, Writing – review & editing. JH: Data curation, Supervision, Writing – review & editing. DS: Data curation, Resources, Supervision, Writing – review & editing. XL: Data curation, Resources, Supervision, Writing – review & editing. YiZ: Data curation, Resources, Supervision, Writing – review & editing. ZH: Funding acquisition, Project administration, Resources, Supervision, Writing – original draft, Writing – review & editing.
