# Supplementary material for: Clinical performance of metagenomic next-generation sequencing for diagnosis of pulmonary Aspergillus infection and colonization
Source: Front Cell Infect Microbiol. 2024 Mar 28;14:1345706. doi: 10.3389/fcimb.2024.1345706 (PMC11007027; doi:10.3389/fcimb.2024.1345706)
Supplement: Supplementary file 2 [file DataSheet_2.docx]

Supplementary Material

## Supplementary Figures


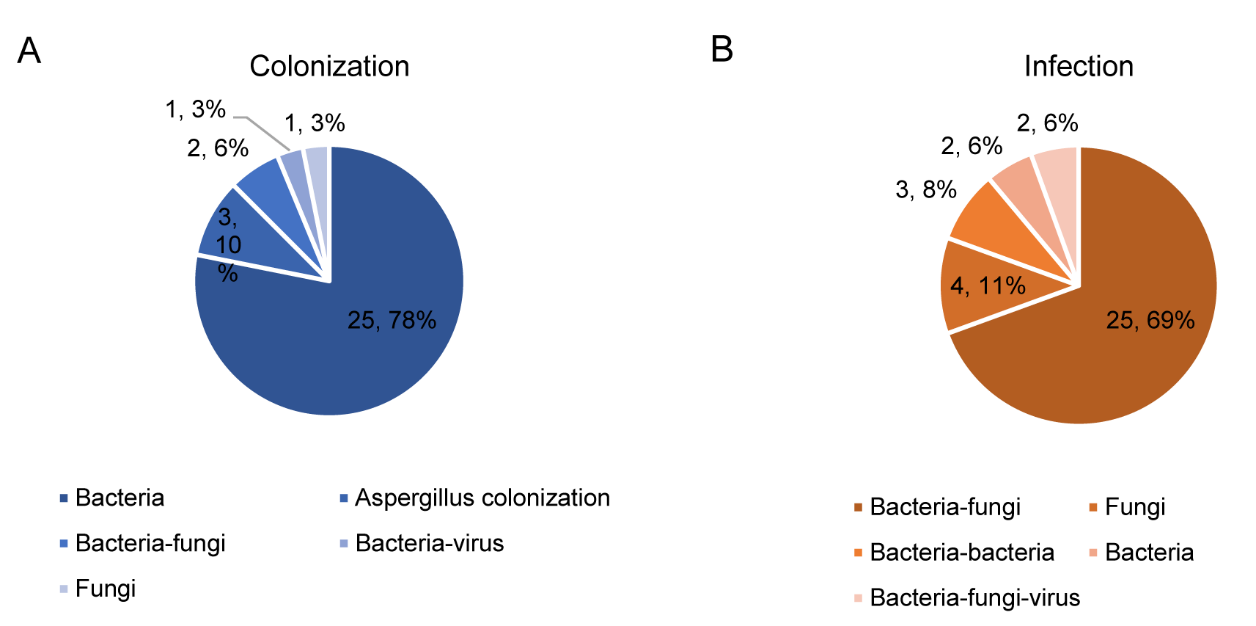


**Supplementary Figure 1.** The infection types of patients with *Aspergillus* colonization (A) and infection (B).

## Supplementary Table

**Supplementary Table1.** The mNGS and conventional microbiological testing (CMT) results of patients with *Aspergillus* infection and colonization. CPA: chronic pulmonary aspergillosis; IPA: invasive pulmonary aspergillosis; CNPA: Chronic necrotizing pulmonary aspergillosis. The red font represents the distribution of *Aspergillus* detected. "/" represents not performing this test.
